# The relationship between swallowing function and clinical parameters in patients with chronic obstructive pulmonary disease

**DOI:** 10.1186/s12890-026-04487-6

**Published:** 2026-07-09

**Authors:** Senay Demir Yazici, Ayşe Kübra Söyler, Hatice Arzu Uçar

**Affiliations:** 1 Department of Physical Medicine and Rehabilitation, Faculty of Medicine, Aydın Adnan Menderes University, Aydın, Turkey; 2 Department of Physiotherapy and Rehabilitation, Faculty of Health Sciences, Aydın Adnan Menderes University, Aydın, Turkey; 3 Department of Chest Diseases, Faculty of Medicine, Adnan Menderes University, Aydın, Turkey

**Keywords:** CAT Score, COPD, Deglutition Disorders, EAT-10, MMRC, RSST

## Abstract

**Background:**

Dysphagia is considered an extrapulmonary manifestation of chronic obstructive pulmonary disease (COPD), and its clinical significance has received increasing attention in recent years. However, data on the association between swallowing function and clinical parameters in COPD remain limited. Therefore, this study aimed to evaluate swallowing function using clinical screening tools in patients with COPD and to assess its relationship with demographic and clinical parameters.

**Methods:**

This cross-sectional study included 60 COPD patients who were followed jointly at the Chest Diseases and Physical Medicine and Rehabilitation outpatient clinics of a tertiary university hospital between April and July 2025. Patients’ swallowing-related parameters were evaluated using the Eating Assessment Tool-10 (EAT-10) and the Repetitive Saliva Swallowing Test (RSST). Physical performance was assessed using the Six-Minute Walk Test (6MWT), respiratory function using spirometric parameters, and symptom severity using the COPD Assessment Test (CAT) and the modified Medical Research Council Dyspnea Scale (mMRC). Data analyses were performed using SPSS.

**Results:**

The mean age of patients was 68.75 ± 6.54 years, and 81.7% were male. According to the EAT-10 screening, the prevalence of self-reported dysphagia was 35%. There was a significant difference in EAT-10 scores between GOLD groups, whereas no difference was observed in RSST counts (*p* = 0.020, *p* = 0.111). Patients with mMRC ≥ 2 had higher EAT-10 scores and lower RSST counts (*p* = 0.043, *p* = 0.024) compared with those with mMRC scores < 2. Patients with CAT scores ≥ 10 had higher EAT-10 scores (*p* = 0.001). Those with recent weight loss also had higher EAT-10 scores and lower RSST counts (*p* = 0.010, *p* = 0.025). No significant correlations were found between swallowing-related parameters and age, BMI, smoking exposure, COPD duration, pulmonary function, or 6-MWT. However, EAT-10 scores showed positive correlations with mMRC (*r* = 0.356, *p* = 0.005) and CAT scores (*r* = 0.530, *p* < 0.001), while RSST counts showed a weak negative correlation with mMRC scores (*r*=-0.282, *p* = 0.029).

**Conclusion:**

Swallowing-related parameters assessed using clinical screening tools were associated with symptom severity (mMRC and CAT) and weight loss in patients with COPD. These findings suggest that screening-based swallowing assessment may provide additional clinical information during clinical follow-up and that screening for dysphagia may be beneficial, particularly in patients with high symptom burden and recent weight loss.

**Trial registration:**

Not applicable.

## Introduction

Chronic obstructive pulmonary disease (COPD) is a chronic disease that causes persistent respiratory symptoms due to airflow obstruction and is associated with many extrapulmonary findings [1]. The disease is typically characterized by recurrent exacerbations, which lead to a decreased functional capacity, impaired quality of life, prolonged hospital stays, and increased healthcare costs [2, 3]. Dysphagia is considered an extrapulmonary symptom of COPD, and in recent years, increasing attention has been focused on this issue [4]. The coordination between swallowing and breathing plays an important role in airway protection, and impairment of this coordination has been reported in patients with COPD [5–7]. In addition, alterations in breathing patterns and impairments in abdominothoracic biomechanics may also contribute to swallowing dysfunction in this population [4, 8, 9]. Previous studies have shown that swallowing typically occurs during the expiratory phase in healthy individuals, whereas swallowing during the inspiratory phase is more common in patients with COPD; this is considered one of the mechanisms associated with an increased risk of aspiration [10]. In addition, dysphagia has been associated with COPD exacerbations. Previous studies have suggested a potential bidirectional relationship between dysphagia and exacerbations, whereby exacerbations may adversely affect swallowing function, while dysphagia may be associated with an increased risk of exacerbations [7, 8, 11]. Considering projections indicating that COPD will rank among the leading causes of mortality worldwide [12], this bidirectional relationship between COPD and dysphagia may contribute to increased mortality and morbidity associated with the disease [7]. This situation explains the increased interest in dysphagia research in COPD patients in recent years. However, the current literature has largely focused on swallowing–breathing coordination and the relationship between dysphagia and exacerbations [4, 7, 13–15]. Although attention has been focused on the clinical significance of dysphagia in COPD, data on how swallowing-related parameters are associated with measurable clinical parameters (such as symptom burden, respiratory function, and exercise capacity) at different stages of the disease are limited. Gonzalez-Lindh et al. demonstrated that swallowing symptoms were particularly associated with high symptom burden and reduced physical capacity, whereas this association was not consistent with objective pulmonary function parameters [16]. These findings suggest that swallowing-related parameters in COPD may be associated with clinical and functional characteristics beyond disease stage, highlighting the need for more detailed evaluations. Identifying factors associated with swallowing dysfunction in COPD patients may be important for the early identification of patients at potential risk of dysphagia. For this purpose, a variety of instrumental and clinical methods are available for the assessment of swallowing function [17, 18]. While videofluoroscopic swallowing study (VFSS) and flexible endoscopic evaluation of swallowing (FEES) are recognized as gold-standard methods for the diagnosis of dysphagia, their routine use may be limited in some clinical settings due to cost, equipment requirements, the need for specialized personnel, and radiation exposure associated with VFSS [17]. Therefore, practical, accessible, and clinically applicable screening tools are of considerable importance. In this context, the Eating Assessment Tool-10 (EAT-10) and the Repetitive Saliva Swallowing Test (RSST) stand out as valid, reliable, and clinically applicable screening tools for dysphagia screening and the assessment of swallowing dysfunction in COPD populations. EAT-10 is one of the most widely used screening tools in dysphagia prevalence studies and is commonly used to evaluate self-reported dysphagia [19, 20]. Studies have also demonstrated good diagnostic performance of EAT-10 compared with instrumental swallowing assessments, as well as strong associations with penetration–aspiration findings [21]. In addition, EAT-10 has been associated with aspiration risk in patients with stable COPD [22]. Similarly, RSST is a simple, objective, and clinically applicable screening tool that evaluates repetitive swallowing performance [23]. Lower RSST counts have been associated with aspiration risk, swallowing dysfunction, and COPD exacerbations [23–25].

Despite the growing body of evidence regarding dysphagia and swallowing dysfunction in COPD, important gaps remain in understanding their relationship with clinical and disease-related characteristics. For this reason, the aim of this study was (i) to investigate the prevalence of self-reported dysphagia in patients with COPD and (ii) to examine the relationship between swallowing-related parameters and demographic and clinical variables.

## Methods

The study was conducted as a collaborative effort between Aydın Adnan Menderes University Faculty of Health Sciences and Faculty of Medicine, and employed a prospective, cross-sectional design. The study received approval from the Non-Interventional Clinical Research Ethics Committee of the Faculty of Medicine, Aydın Adnan Menderes University (06 March 2025; protocol no. 2025/63), and was performed in compliance with the Declaration of Helsinki. Written informed consent was obtained from all participants. The study was carried out between April and July 2025 at the Pulmonary Diseases outpatient clinic of our university hospital. Patients with COPD who were followed on an outpatient basis and referred to the Physical Medicine and Rehabilitation (PM&R) department for pulmonary rehabilitation were screened for eligibility. Those who met the inclusion criteria and volunteered to participate were enrolled in the study. Inclusion criteria included age ≥ 40 years, a confirmed diagnosis of COPD in a stable clinical phase, and availability of a pulmonary function test (PFT) performed within the preceding three months. Individuals were excluded if they had an active infection, coexisting chronic pulmonary conditions, a history of unstable angina or myocardial infarction within the preceding six months, or any additional disorder with the potential to impair swallowing function, including neurological diseases (e.g., stroke, Parkinson’s disease, dementia, or neuromuscular disorders), head and neck cancer, or previous head and neck surgery [22, 25]. The presence of these conditions was assessed based on documented physician diagnoses recorded in the hospital electronic medical records and patient files, together with the medical history obtained during the outpatient evaluation.

The COPD Assessment Test (CAT), the Modified Medical Research Council Dyspnea Scale (mMRC), and PFT were administered in the pulmonology outpatient clinic. PFT results were used to stage patients, and they were also grouped according to the Global Initiative for Chronic Obstructive Lung Disease (GOLD 2023) classification. Following these assessments, patients were referred to the PM&R outpatient clinic for further examination and rehabilitation. In the outpatient clinic, a PM&R physician experienced in pulmonary rehabilitation recorded the patients’ demographic data and administered the 6-Minute Walk Test (6MWT). The swallowing-related assessments including EAT-10 and RSST were performed by a physiotherapist with eight years of clinical experience in dysphagia assessment and management. To reduce the potential impact of disease fluctuation, all assessments were performed on the same day.

### Clinical and demographic characteristics of participants

Gender, age, weight, height, smoking exposure, disease duration, comorbidities, and steroid use were recorded. Information regarding weight loss was obtained from medical records. Current body weight was compared with that recorded approximately one year earlier at a follow-up visit in the Pulmonology outpatient clinic, and a loss of ≥ 5% of baseline body weight was considered weight loss.

### Modified medical research council (mMRC) dyspnea scale

The mMRC was used to assess dyspnea severity. This tool is a widely used subjective assessment tool that rates breathlessness during daily activities on a scale of 0 to 4. A higher score indicates more severe breathlessness [26, 27].

### COPD assessment test (CAT)

The CAT was applied to measure the burden of symptoms related to COPD and their impact on everyday life. This tool consists of eight questions related to symptoms, sleep, confidence, and exercise limitations. The total possible score extends to 40, with each item contributing between 0 and 5 points. Higher scores indicate a greater symptom burden and a greater impact of COPD on daily life [28, 29].

### Pulmonary function test (PFT)

FEV₁, FVC, and FEV₁/FVC values were recorded from pulmonary function test results to evaluate the patients’ respiratory status and determine the disease stage. A fixed FEV₁/FVC ratio of < 0.70 was considered indicative of airflow limitation and COPD [30].

### Global initiative for chronic obstructive lung disease (GOLD) 2023 classification

COPD staging and classification were performed based on FEV₁ and symptom severity according to the GOLD 2023 criteria. Under this system, disease stages were defined based on the ratio of FEV₁ to the predicted value, with Stage 1 classified as ≥ 80%, Stage 2 as 50–79%, Stage 3 as 30–49%, and Stage 4 as < 30%. Patients were divided into groups A (low symptom/low exacerbation risk), B (high symptom/low exacerbation risk), and E (high exacerbation risk) based on symptom severity and annual exacerbation frequency [31].

### 6-Minute walk test (6MWT)

The 6MWT was employed to measure exercise tolerance. Patients were asked to walk the longest possible distance in six minutes along a flat 30-meter corridor. The total distance covered at the end of the test was recorded in meters (m) [32].

### Eating assessment tool-10 (EAT-10)

The EAT-10 was utilized for dysphagia screening and to determine symptom severity. The EAT-10 is a quick, self-administered, easy-to-understand, symptom-specific screening tool for the subjective assessment of swallowing-related symptoms. The tool consists of 10 items scored on a 5-point Likert scale, where 0 indicates “no problem” and 4 represents a “very serious problem.” Cumulative scores range from 0 to 40, with higher values reflecting greater symptom severity [33, 34]. Although various thresholds have been suggested, a recent systematic review and meta-analysis recommended a cutoff value of 3 because of its better diagnostic accuracy [21]. Consequently, in the present study, patients with an EAT-10 score ≥ 3 were classified as having self-reported dysphagia [20].

### Repetitive saliva swallowing test (RSST)

RSST was used to evaluate repetitive swallowing performance. This is a simple and clinically applicable test that evaluates a patient’s ability to swallow repeatedly without the administration of an external bolus [23, 24]. During the assessment, the participant was instructed to repeatedly swallow saliva as many times as possible over a 30-second period while seated with the head in a neutral position. The evaluator recorded the number of swallows by palpating the larynx [24]. In the present study, RSST count was used as a functional indicator of swallowing performance to investigate its relationship with the clinical characteristics of COPD.

### Statistical analysis

The statistical power of the study was estimated using a post-hoc analysis performed with G*Power software (version 3.1.9.7). The analysis was based on differences in EAT-10 scores between patients with CAT scores < 10 (*n* = 17) and those with CAT scores ≥ 10 (*n* = 43). Given an observed effect size (Cohen’s d) of 0.966 and a significance level of 0.05, the achieved statistical power (1 − β) was calculated to be 91.2%.

Statistical evaluations were conducted utilizing IBM SPSS Statistics software, version 25.0 (IBM Corp., Armonk, NY, USA). To determine the distribution characteristics of the continuous data, the Kolmogorov–Smirnov test was employed. Given that most variables did not exhibit a normal distribution, non-parametric analytical approaches were employed. Descriptive statistics included mean ± standard deviation for data with a normal distribution and median (25th–75th percentiles) for data not following a normal distribution. Categorical data were detailed as counts (n) and percentages (%). The Mann–Whitney U test was utilized to compare two independent groups, while the Kruskal–Wallis test served for comparisons across multiple groups. Relationships between demographic, clinical, and functional characteristics and EAT-10 scores and RSST counts were explored using Spearman’s rank correlation coefficients. Statistical significance was defined as a p value below 0.05.

## Results

A total of 60 patients with stable COPD were enrolled in the study. The majority of the cohort was male (81.7%), and the mean age was 68.75 ± 6.54 years. Based on the GOLD classification, 13 patients (21.7%) were assigned to Group A, 7 (11.7%) to Group B, and 40 (66.7%) to Group E. Detailed clinical and demographic characteristics of the study population are summarized in Table 1. According to the EAT-10 screening, the prevalence of self-reported dysphagia was 35% among participants.

**Table 1:** Clinical and demographic characteristics of patients with COPD

Variables	
Gender, *n* (%)	
Male	49 (81.7%)
Female	11 (18.3%)
Age	68.75 ± 6.54
BMI, kg/m²	25.59 ± 4.87
Smoking habits of participants, n (%)	
Active smokers	28 (46.7%)
Former smokers	26 (43.3%)
Non-smoker	6 (10%)
Smoking exposure, pack-years	49.5 (30.75–60)
Group Classification n (%)	
Group A	13 (21.7%)
Group B	7 (11.7%)
Group E	40 (66.7%)
Disease stage based on FEV₁, n(%)	
Stage 1 FEV₁ ≥80% predicted	6 (10%)
Stage 2 FEV₁ 50–79% predicted	21 (35%)
Stage 3 FEV₁ 30–49% predicted	27 (45%)
Stage 4 FEV₁ <30% predicted	6 (10%)
Duration of COPD, years	7 (3.75–15.25)
Steroid use, n(%)	
Yes	46 (76.7%)
No	14 (23.3%)
Presence of comorbidity, n(%)	
None	17 (28.3%)
≥ 1 comorbidity	43 (71.7%)
Co-morbidities, n (%)	
Hypertension	23(38.3%)
Diabetes mellitus	12(20%)
Cardiovascular disease	18(30%)
Hypothyroidism	6(10%)
Rheumatologic disease	2(3.3%)

Continuous data are summarized according to their distribution as mean ± standard deviation or median (interquartile range). Categorical variables are reported as n (%)

*Abbreviations*: *COPD* Chronic obstructive pulmonary disease, *FEV1* Forced expiratory volume in 1 second

**Fig. 1:**
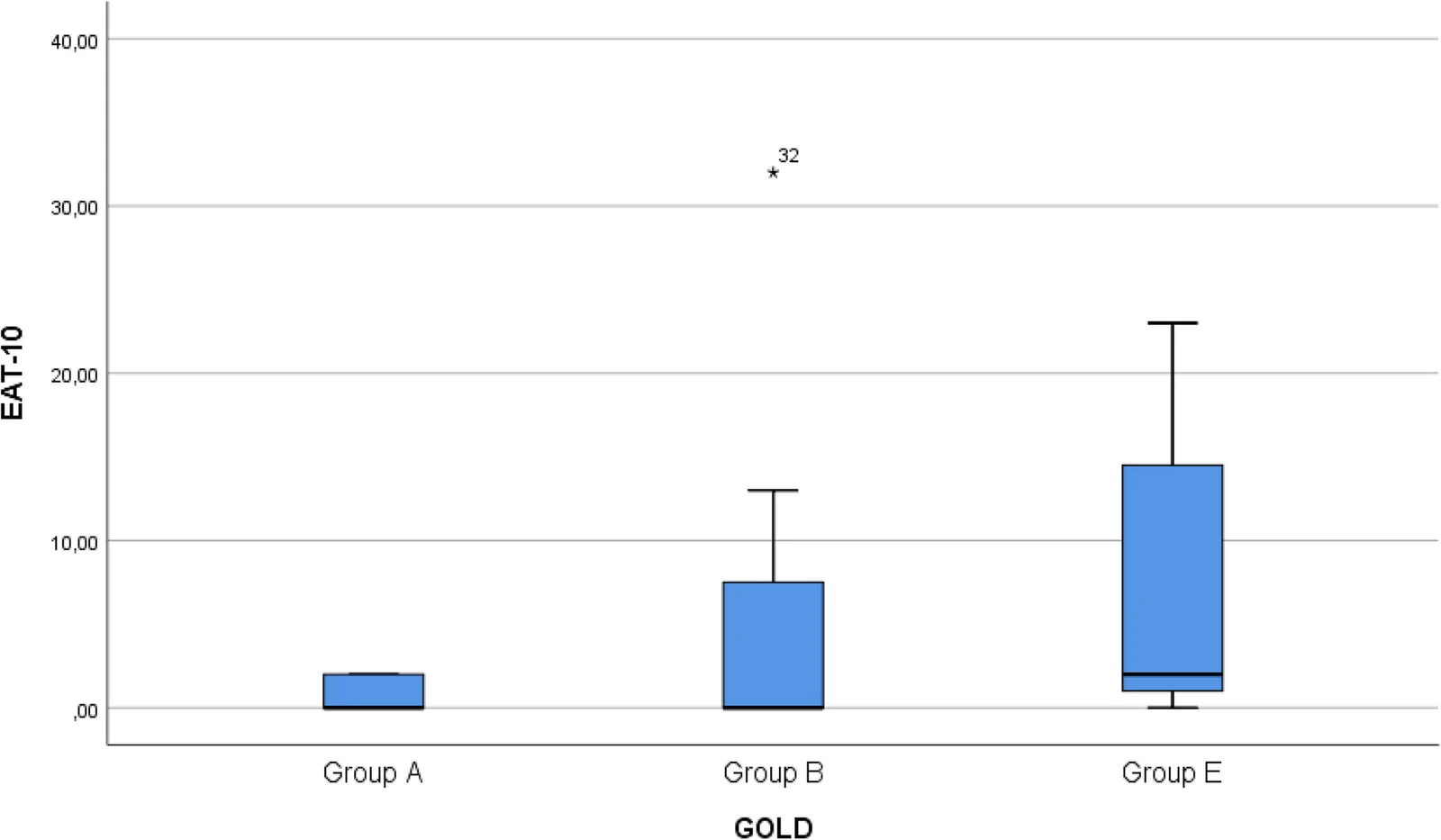
Box-and-whisker plots illustrating the distribution of EAT-10 scores across GOLD classification groups. A significant difference was observed between Group A and Group E (*p* = 0.005). Abbreviations: EAT-10, Eating assessment tool-10; GOLD, Global initiative for chronic obstructive lung disease

**Fig. 2:**
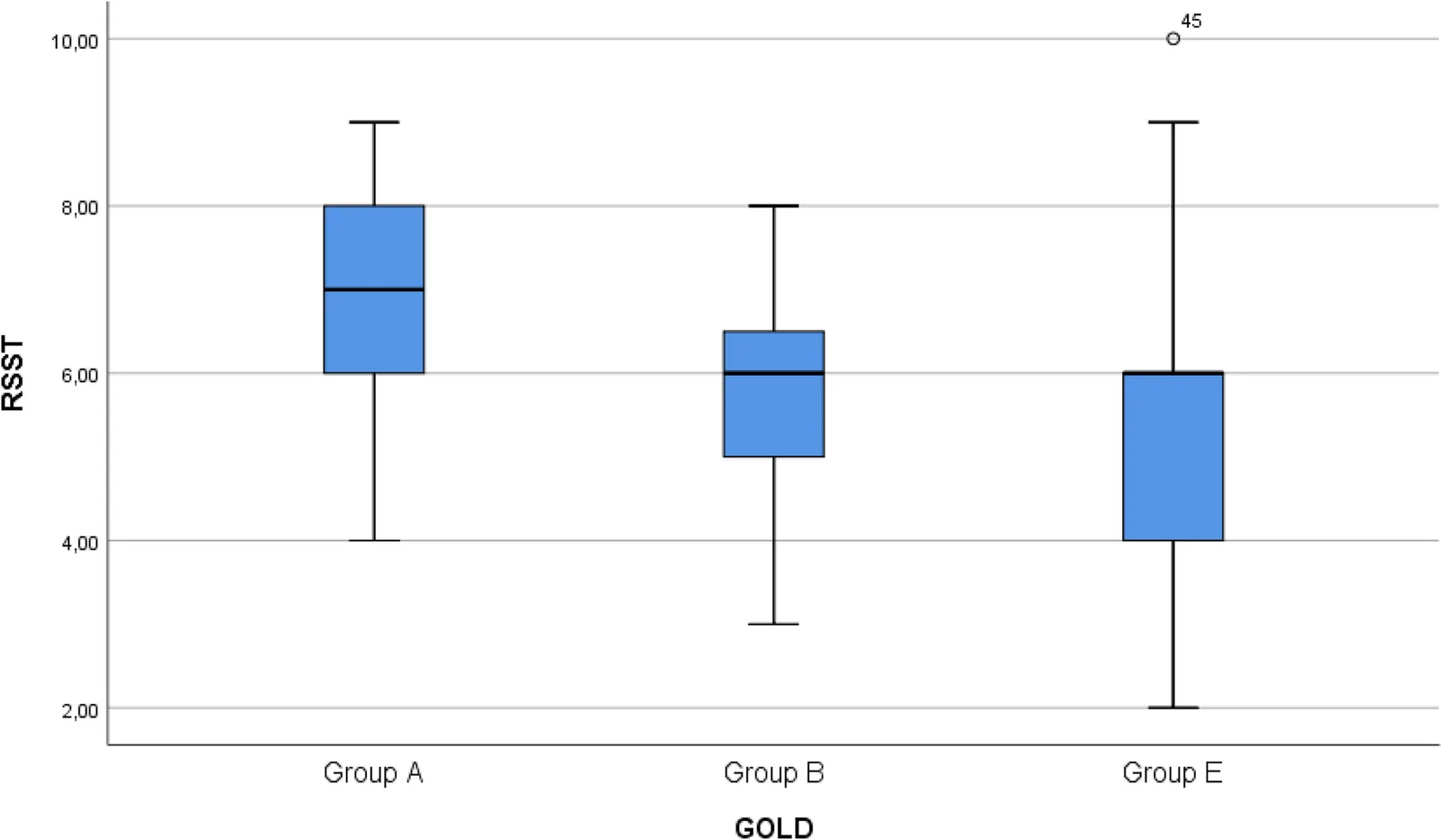
Box-and-whisker plots illustrating the distribution of RSST counts across GOLD classification groups. A significant difference was observed between Group A and Group E (*p* = 0.037). Abbreviations: GOLD, Global initiative for chronic obstructive lung disease; RSST, Repetitive saliva swallowing test

While there was a significant difference in EAT-10 scores between GOLD groups, there was no difference in the RSST counts (*p* = 0.020, *p* = 0.111). When comparing the groups, only patients in Group E had significantly higher EAT-10 scores and significantly lower RSST counts compared to those in Group A (*p* = 0.005, *p* = 0.037). No significant difference was found between Groups A and B (*p* = 0.630, *p* = 0.260) or between Groups B and E (*p* = 0.289, *p* = 0.679) in terms of swallowing-related parameters (Table 2; Figs. 1 and 2). In contrast, no significant differences in EAT-10 scores or RSST counts were observed when patients were grouped according to FEV₁ stages (*p* = 0.350, *p* = 0.542) (Table 2). No significant correlations were found between EAT-10 scores or RSST counts and age, BMI, smoking exposure, COPD duration, and 6MWT distance; furthermore, no significant relationship was found between steroid use or comorbidity status and swallowing-related parameters (*p* > 0.05). There was no difference in EAT-10 scores between genders (*p* = 0.085), but RSST counts were lower in women than in men (*p* = 0.019). Patients who had lost weight in the past year were found to have significantly higher EAT-10 scores and lower RSST counts compared to those who had not lost weight (*p* = 0.010, *p* = 0.025) (Tables 2 and 3).

**Table 2:** EAT-10 score and repetitive saliva swallowing test comparisons according to clinical and disease severity subgroups in COPD

Variables	*n*	EAT-10	*p*	RSST	*p*
Gender					
Male	49	2 (0–4.5)	0.085	6 (5–7)	**0.019**
Female	11	9 (1–15)		5 (3–6)	
Steroid use					
Yes	46	2 (0–13.25)		6 (5–7)	
No	14	1.5 (0–3.25)	0.520	5.5 (4–7)	0.486
Comorbidities					
Yes	43	2 (0–13)		6 (4–7)	
No	17	1 (0–2)	0.068	6 (5–7.5)	0.407
Weight loss					
Yes	21	9 (0.5–19.5)		5 (3.5–6)	**0.025**
No	39	1 (0–2)	**0.010**	6 (5–7)	
Between COPD groups	60	2 (0–9.75)	**0.020**	6 (5–7)	0.111
Group A-Group B	13 − 7	0 (0–2)−0 (0–13)	0.630	7 (5.5–8)- 6 (5–7)	0.260
Group A-Group E	13–40	0 (0–2)−2 (1–14.75)	**0.005**	7 (5.5–8)- 6 (4–6)	**0.037**
Group B-Group E	7–40	0 (0–13)−2 (1–14.75)	0.289	6 (5–7)- 6 (4–6)	0.679
mMRC score ≥ 2	46	2 (0–14.75)		6 (4–6)	
mMRC score < 2	14	1 (0–2)	**0.043**	6.5 (5.75–8.25)	**0.024**
CAT score ≥ 10	43	2 (0–15)		6 (5–6)	
CAT score < 10	17	0 (0–1.5)	**0.001**	6 (4.5–7.5)	0.366
Between FEV₁ stage groups	60	2 (0–9.75)	0.350	6 (5–7)	0.542
Stage 1 and 2	6–21	1 (0–1)−2 (0.5–12.5)	0.062	6(4.75–6.5)- 6(5–7.5)	0.635
Stage 1 and 3	6–27	1 (0–1)−2 (0–14)	0.288	6 (4.75–6.5)- 6 (4–7)	0.755
Stage 1 and 4	6–6	1 (0–1)−1.5 (0–8)	0.310	6(4.75–6.5)- 5.5(4–6)	0.388
Stage 2 and 3	21–27	2 (0.5–12.5)−2 (0–14)	0.523	6 (5–7.5)- 6 (4–7)	0.296
Stage 2 and 4	27 − 6	2(0.5–12.5)−1.5 (0–8)	0.592	6 (5–7.5)- 5.5 (4–6)	0.182
Stage 3 and 4	21 − 6	2 (0–14)−1.5 (0–8)	0.942	6 (4–7)- 5.5 (4–6)	0.597

Numerical data are expressed as median (interquartile range, 25th–75th percentiles). Statistical significance across multiple groups was determined using the Kruskal–Wallis test. For specific dual-group comparisons (e.g., A vs. B or individual disease stages), the Mann–Whitney U-test was employed. Bold values indicate statistically significant results (*p* < 0.05)

*Abbreviations*: *CAT* COPD Assessment test, *COPD* Chronic obstructive pulmonary disease, *EAT-10* Eating assessment tool-10, *mMRC* Modified medical research council, *RSST* Repetitive saliva swallowing test

**Table 3:** Correlations between EAT-10 scores, repetitive saliva swallowing test and demographic, clinical, and functional parameters in patients with COPD

	*n*	EAT-10 (*r*)	EAT-10 (*p*)	RSST (*r*)	RSST (*p*)
Age, years	60	-0.156	0.235	0.101	0.443
BMI, kg/m²	60	−0.132	0.316	0.002	0.988
Smoking exposure, pack-years	60	0.100	0.449	0.032	0.808
Duration of COPD, years	60	0.117	0.398	0.031	0.823
mMRC	60	0.356	**0.005**	−0.282	**0.029**
CAT	60	0.530	**< 0.001**	−0.205	0.117
FEV_1_ (%)	60	−0.058	0.667	0.164	0.219
FVC (%)	60	−0.079	0.552	0.139	0.295
FEV_1_/FVC	60	−0.020	0.883	0.040	0.763
Six-Minute Walk Test (meters)	60	−0.143	0.277	−0.032	0.806
Six-Minute Walk Test (%)	60	−0.042	0.750	0.015	0.907

Associations were assessed using Spearman’s rank correlation analysis. *r* denotes the correlation coefficient and *p* indicates the level of statistical significance. A positive r reflects a direct relationship between variables, whereas a negative *r* reflects an inverse relationship. Bold values indicate statistically significant results (*p* < 0.05)

*Abbreviations*: *6MWT* Six-minute walk test, *BMI* Body mass index, *CAT* COPD assessment test, *COPD* Chronic obstructive pulmonary disease, *EAT-10* Eating assessment tool-10, *FEV1* Forced expiratory volume in 1 second, *FVC* Forced vital capacity, *mMRC* Modified medical research council dyspnea scale, *RSST* Repetitive saliva swallowing test

**Fig. 3:**
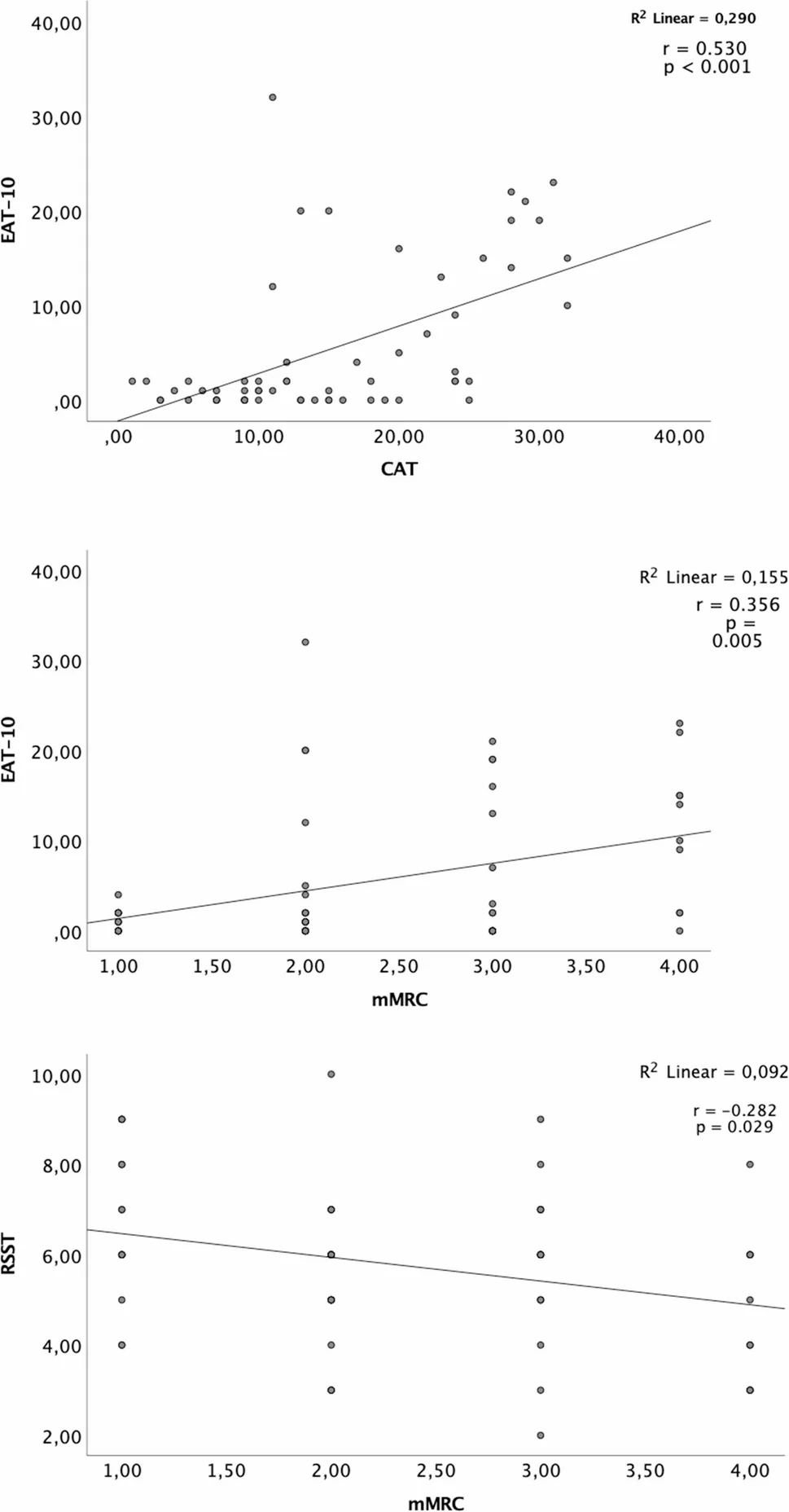
Scatter plots illustrating the associations between swallowing-related parameters and mMRC and CAT scores in patients with COPD. Abbreviations: CAT, COPD Assessment test; COPD, chronic obstructive pulmonary disease; EAT-10, Eating assessment tool-10; mMRC, modified Medical research council dyspnea scale; RSST, Repetitive saliva swallowing test

When patients with an mMRC score ≥ 2 were compared with those with a score < 2, a significant difference was found in both EAT-10 scores (*p* = 0.043) and RSST counts (*p* = 0.024). When patients with a CAT score ≥ 10 were compared with those with a score < 10, a significant difference was found only in EAT-10 scores (*p* = 0.001) (Table 2). Additionally, significant correlations were found between mMRC scores and swallowing-related parameters, including a positive correlation with EAT-10 scores (*r* = 0.356, *p* = 0.005) and a negative correlation with RSST counts (*r*=−0.282, *p* = 0.029). A moderate positive correlation was found between CAT and EAT-10 scores (*r* = 0.530, *p* < 0.001) (Table 3). Scatter plots demonstrating the correlations between swallowing-related parameters (EAT-10 and RSST) and clinical symptom scores (CAT and mMRC) are presented in Fig. 3.

## Discussion

To our knowledge, this is the first study to investigate swallowing-related parameters and the screening-based prevalence of dysphagia in patients with COPD in the Turkish population. In the present study, the prevalence of self-reported dysphagia in patients with stable COPD was found to be 35%. Our findings suggest that in stable COPD patients, age, BMI, presence of comorbidities, steroid use, smoking exposure, disease duration, and functional exercise capacity were not associated with dysphagia symptom severity or repetitive swallowing performance. In contrast, weight loss, dyspnea severity, disease symptom burden, and GOLD group classification were associated with swallowing-related parameters, including dysphagia symptom severity and repetitive swallowing performance.

Previous studies have shown that dysphagia is common in patients with COPD, with the reported prevalence ranging widely across the literature (17–85%) [16, 35–37]. This variability has been attributed to differences in sample characteristics (stable period vs. exacerbation period, etc.) and the assessment methods used [2, 15]. Some studies used screening-based assessments, including dysphagia questionnaires and clinical swallowing tests, whereas others used instrumental swallowing assessments. A study conducted in Sweden reported that 65% of stable COPD patients had dysphagia based on questionnaire assessment, while a recent meta-analysis reported a pooled prevalence of approximately 32.7% for oropharyngeal dysphagia in COPD [36, 37]. The prevalence of self-reported dysphagia in our study was 35%, which appears to be generally consistent with the prevalence ranges reported in the literature. Nevertheless, the relatively small sample size and the use of screening-based methods should be considered when interpreting these prevalence findings. Therefore, further studies including larger samples and instrumental swallowing assessments may help to better clarify the prevalence of dysphagia in COPD.

It has been reported that dysphagia in COPD patients is a multifactorial condition and has been associated with factors such as weakness in the swallowing muscles, impaired swallowing-breathing coordination, dyspnea, low physical capacity, and xerostomia [22, 37, 38]. Our study found that age was not associated with dysphagia symptom severity or repetitive swallowing performance; however, repetitive swallowing performance was higher in men. Although no statistically significant difference was found between gender and dysphagia symptom severity, women tended to report higher dysphagia symptom severity. A previous study conducted in stable COPD patients also reported no significant difference in terms of gender [36], and the literature reports higher RSST values in men. These findings are generally consistent with previous reports regarding gender-related differences in swallowing-related parameters in COPD [24, 36]. On the other hand, although advanced age is considered a risk factor for dysphagia, findings are inconsistent in COPD; while one study associated ≥ 74 years of age with high risk, no association between age and dysphagia was found in patients with acute exacerbations of COPD [7, 13]. Given the inconsistent findings in the literature regarding the association between age and swallowing-related parameters in COPD, further studies including patients with different disease severities may help to clarify this relationship.

In COPD, cachexia is an important extrapulmonary finding associated with increased mortality risk, disease burden, and reduced quality of life [39]. In this context, BMI and weight loss are considered important clinical indicators in COPD. No significant relationship was found between BMI and swallowing-related parameters; however, patients with weight loss showed increased dysphagia symptom severity and a decrease in repetitive swallowing performance. Previous studies have also reported that BMI may not be an appropriate indicator for determining dysphagia in either the exacerbation period or in stable COPD [13, 16]. Considering previous recommendations suggesting that weight loss may provide more clinically meaningful information than BMI in COPD [39], together with the findings of the present study, weight loss may be more closely related to dysphagia in COPD.

Patients with COPD experience a decrease in functional capacity independent of physiological impairment. The 6MWT is widely used to assess functional capacity and is considered an important indicator of exacerbations in COPD patients [40]. Research examining the association between dysphagia and functional capacity in individuals with COPD remains scarce. In this context, only one study by Gonzalez-Lindh et al. has investigated the association between swallowing function and the 30-meter walking test in stable COPD patients. A significant relationship was found between these two variables, and it was reported that swallowing dysfunction was associated with lower physical capacity [16]. However, no correlation was found between the 6-minute walk test and swallowing-related parameters in the present study, which may suggest that functional capacity alone may not sufficiently explain swallowing function in COPD.

One of the main findings of this study is that increased dyspnea severity is associated with increased dysphagia symptom severity and reduced repetitive swallowing performance. A positive correlation was found between the mMRC score, which measures dyspnea perception, and the EAT-10 score, which indicates dysphagia symptom severity. Similarly, a moderate positive correlation was also identified between the CAT score, which reflects symptom burden, and the EAT-10 score. Additionally, the presence of a weak negative correlation between the mMRC score and the RSST count, which reflects repetitive swallowing performance, may suggest that repetitive swallowing performance is negatively affected by increasing dyspnea severity. These findings appear to be consistent with the reported bidirectional interaction between dysphagia and dyspnea. It has been previously reported that swallowing function may be affected in respiratory system diseases and that dysphagia may increase dyspnea through changes in respiratory patterns [4–8]. In addition, one study reported that 74% of COPD patients experienced dyspnea during feeding [41]. Furthermore, it has been reported that swallowing performance, as assessed by RSST, is affected by many factors, such as the excitability of the swallowing reflex, the speed and coordination of swallowing movements, and muscle strength [42]. Our findings are consistent with the results of the study conducted by Gonzalez-Lindh et al., which found that swallowing symptoms were associated with high levels of dyspnea and increased symptom burden in patients with COPD [16]. When these data are evaluated together, it may be useful to consider swallowing-related parameters during the clinical evaluation of COPD patients with increased dyspnea severity and symptom burden.

In this study, no significant relationship was found between functional parameters represented by FEV₁, FVC, and FEV₁/FVC and swallowing-related parameters. Findings regarding this subject vary; while some studies report no association between pulmonary function and swallowing-related parameters, others have reported that swallowing function is affected in COPD with moderate to severe airflow limitation [16, 36]. It has been suggested that these differences may stem from differences in the age and symptom profiles of the study populations, as well as from the methods used to assess dysphagia [16]. In this sense, our findings suggest that spirometric parameters alone may not sufficiently reflect swallowing function in COPD.

Another important finding of this study is that GOLD group classification was associated with swallowing-related parameters in our cohort, with significant differences observed between Group A and Group E. Patients in Group E, who had the highest disease severity and risk of exacerbation, had higher EAT-10 scores and lower RSST counts compared to those in Group A, who had the lowest symptom burden. The lack of significant differences between Groups A and B and between Groups B and E suggests that the relationship between GOLD group classification and swallowing-related parameters may not be linear. However, these findings should be interpreted with caution, as the relatively small number of participants in Group B (*n* = 7) may have limited the statistical power of comparisons involving this subgroup. The significant differences observed between Groups A and E may indicate that swallowing-related impairments become more apparent in patients with greater symptom burden and exacerbation risk. In the literature, dysphagia has been reported to be more prevalent, particularly in GOLD groups with high symptom burden and exacerbation risk [16]. Parallel to this, it has been reported that RSST counts are significantly lower in patients classified as GOLD Group E and that this test may be considered a potential predictor of exacerbation risk [43]. Taken together, these findings indicate that patients classified as GOLD Group E have less favorable swallowing-related outcomes compared to those in lower-risk GOLD groups. Therefore, dysphagia should not be overlooked during the clinical follow-up of these patients, and swallowing function should be considered and evaluated as part of their clinical assessment.

This study has several limitations that should be considered when interpreting the findings. First, the study was conducted at a single tertiary center and included a relatively small sample, with a predominance of patients in the GOLD E group, which may limit the generalizability of the results. In addition, the relatively small number of participants in GOLD Group B should be considered when interpreting subgroup comparisons. Second, swallowing function was evaluated using screening-based tools (EAT-10 and RSST), and no instrumental swallowing assessment such as FEES or VFSS was performed. Therefore, the findings should be interpreted as reflecting self-reported dysphagia symptoms and repetitive swallowing performance rather than confirmed dysphagia. Third, the cross-sectional design precludes conclusions regarding causality. In addition, no multivariable analyses were performed; therefore, the observed associations should not be interpreted as independent relationships. Lastly, although several conditions known to affect swallowing function were excluded, gastroesophageal reflux disease was not specifically assessed. To better understand the relationship between swallowing function and clinical characteristics in COPD, future multicenter prospective studies incorporating instrumental swallowing assessments are needed.

## Conclusion

This study revealed that self-reported dysphagia is present in more than one-third of patients with stable COPD. Weight loss, dyspnea severity, symptom burden, and GOLD group classification were associated with swallowing-related parameters. These findings highlight the importance of considering swallowing function during the clinical evaluation of patients with COPD, particularly those with greater symptom burden, a history of weight loss, and those classified as GOLD Group E.

## Data Availability

The datasets generated and/or analysed during the current study are not publicly available due to participant confidentiality and institutional data protection policies but are available from the corresponding author on reasonable request.
